# Study of Electrical and Dielectric Behaviors of Copper-Doped Zinc Oxide Ceramic Prepared by Spark Plasma Sintering for Electronic Device Applications

**DOI:** 10.3390/nano14050402

**Published:** 2024-02-22

**Authors:** Majdi Benamara, Kais Iben Nassar, Pedro Rivero-Antúnez, Manel Essid, Silvia Soreto Teixeira, Shanyu Zhao, Albert Serrà, Luis Esquivias

**Affiliations:** 1 Laboratory for Building Energy Materials and Components, Swiss Federal Laboratories for Materials Science and Technology (Empa), 8600 Dübendorf, Switzerland; 2 I3N-Aveiro, Department of Physics, University of Aveiro, 3810-193 Aveiro, Portugal; kais.nassar@ua.pt (K.I.N.); silvia.soreto@ua.pt (S.S.T.); 3 CICECO—Aveiro Institute of Materials, Department of Chemistry, University of Aveiro, Campus Universitário de Santiago, 3810-193 Aveiro, Portugal; 4 Departamento de Física de la Materia Condensada, Universidad de Sevilla, 41012 Sevilla, Spain; privero@us.es (P.R.-A.); luisesquivias@us.es (L.E.); 5 Simidea R&D, European Business and Innovation Centre of Cartagena, 30353 Cartagena, Spain; 6 Department of Chemistry, College of Science, King Khalid University, Abha 61413, Saudi Arabia; maiseed@kku.edu.sa; 7 Thin Films and Nanostructures Electrodeposition Group (GE-CPN), Department of Materials Science and Physical Chemistry, University of Barcelona, 08028 Barcelona, Spain; a.serra@ub.edu

**Keywords:** copper-doped ZnO, Spark Plasma Sintering, electrical conductivity, impedance spectroscopy, activation energy, electronic device applications

## Abstract

In this study, Cu-doped ZnO aerogel nanoparticles with a 4% copper concentration (Cu4ZO) were synthesized using a sol–gel method, followed by supercritical drying and heat treatment. The subsequent fabrication of Cu4ZO ceramics through Spark Plasma Sintering (SPS) was characterized by X-ray diffraction (XRD), field-emission gun scanning electron microscopy (FE-SEM) equipped with EDS, and impedance spectroscopy (IS) across a frequency range of 100 Hz to 1 MHz and temperatures from 270 K to 370 K. The SPS–Cu4ZO sample exhibited a hexagonal wurtzite structure with an average crystallite size of approximately 229 ± 10 nm, showcasing a compact structure with discernible pores. The EDS spectrum indicates the presence of the base elements zinc and oxygen with copper like the dopant element. Remarkably, the material displayed distinct electrical properties, featuring high activation energy values of about 0.269 ± 0.021 eV. Complex impedance spectroscopy revealed the impact of temperature on electrical relaxation phenomena, with the Nyquist plot indicating semicircular arc patterns associated with grain boundaries. As temperature increased, a noticeable reduction in the radius of these arcs occurred, coupled with a shift in their center points toward the axis center, suggesting a non-Debye-type relaxation mechanism. Dielectric analyses revealed a temperature-driven evolution of losses, emphasizing the material’s conductivity impact. Non-Debye-type behavior, linked to ion diffusion, sheds light on charge storage dynamics. These insights advance potential applications in electronic devices and energy storage.

## 1. Introduction

In recent years, significant strides have been made in enhancing the electrical and dielectric performance of n-type ZnO-based ceramics for electronic devices [1,2,3]. Innovative strategies, including Group 3 element doping, polymer insertion, and morphology modification, have prompted deeper explorations into the potential of specific dopants—copper (Cu), Aluminum (Al), Gallium (Ga), and Indium (In)—in ZnO [4,5,6,7]. These dopants, with ionic radii closely matching that of Zn, hold the capacity to significantly improve the electrical properties by modulating the oxide’s band gap. The substitution of Cu into the zinc site has particularly demonstrated advantages in enhancing electrical and dielectric properties through nanostructuring, making Cu-doped ZnO a promising candidate for high-performance electronic materials [4,8,9]. While advancements have been notable, a key challenge in the realm of electronic applications lies in processing nanostructured ceramics from nanopowders [10]. 

To address this challenge, our research focuses on the synthesis and characterization of copper-doped ZnO nanoparticles and ceramics using controlled sol–gel methods and Spark Plasma Sintering (SPS) at a consistent temperature of 1000 °C. This investigation aims to unravel the intricate interplay between synthesis parameters and resulting electronic performance, contributing valuable insights to the ongoing advancements in the design and application of ZnO-based electronic materials [11,12]. Despite various studies exploring SPS sintering of ZnO, the need for innovative approaches that achieve both high relative densities and controlled grain sizes remains [13,14]. Our research seeks to bridge this gap by extending into controlled sol–gel methods and employing SPS to maintain a consistent temperature. By comprehensively investigating copper-doped ZnO materials, we aim to shed light on the synthesis intricacies and their impact on the electronic performance, providing essential contributions to the evolving landscape of electronic device applications [15,16]. Our approach involves proposing a method to simultaneously achieve low thermal conductivity and a high thermoelectric power factor using controlled dopant concentration and coherent homoepitaxial interfaces. Several researchers have harnessed the distinct advantages of Spark Plasma Sintering (SPS) to achieve finer grain sizes and a more uniform distribution compared to conventional sintering processes under similar conditions. Kharchouche et al. demonstrated a consistently homogeneous microstructure in ZnO varistor ceramics using SPS across a temperature range of 900 to 1200 °C, employing various heating rates. However, despite this achievement, the electrical properties showed only marginal improvement, with the nonlinear coefficient reaching approximately 23 [17]. In a separate study, Kougo et al. observed that pure ZnO could be effectively densified through SPS, resulting in an increase in grain size from 1 to 100 μm as the sintering temperature elevated from 450 °C to 1200 °C [18]. The demonstrated success with interface carrier scattering highlights the potential for substantial power factor enhancement, paving the way for significant advancements in electronic applications [19,20].

This study represents a significant step forward in the field by undertaking a comprehensive exploration of copper-doped zinc oxide (Cu4ZO) materials, with a specific focus on the synthesis and detailed characterization of both nanoparticles and ceramics. The nanoparticle synthesis employed a meticulous sol–gel method, ensuring the precise incorporation of copper dopants into the zinc oxide matrix. Subsequent supercritical drying using ethyl alcohol (EtOH) and controlled heat treatment resulted in the production of well-defined Cu-doped ZnO nanoparticles (Cu4ZO). Transitioning from nanoparticles to ceramics was achieved through the highly effective SPS technique, subjecting the Cu4ZO nanopowder to a consistent temperature of 1000 °C, maintaining a nitrogen gas atmosphere, and applying a uniaxial pressure of 50 MPa for 3 min. The resulting pellet, denoted as SPS–Cu4ZO, underwent a thorough examination of its structural, morphological, electrical, and dielectric properties. The structural analysis, including X-ray diffraction (XRD), and the morphological/chemical composition assessed through scanning electron microscopy (SEM) equipped with EDS were studied. The electrical and dielectric behaviors were studied by impedance spectra (IS). The knowledge gained not only advances the understanding of Cu4ZO materials but also lays the groundwork for targeted optimizations, fostering innovation in the design and application of zinc-oxide-based electrical device materials for enhanced efficiency and tailored functionalities.

## 2. Materials and Methods

### 2.1. Cu-Doped ZnO Nanoparticle Preparation

**Figure 1:**
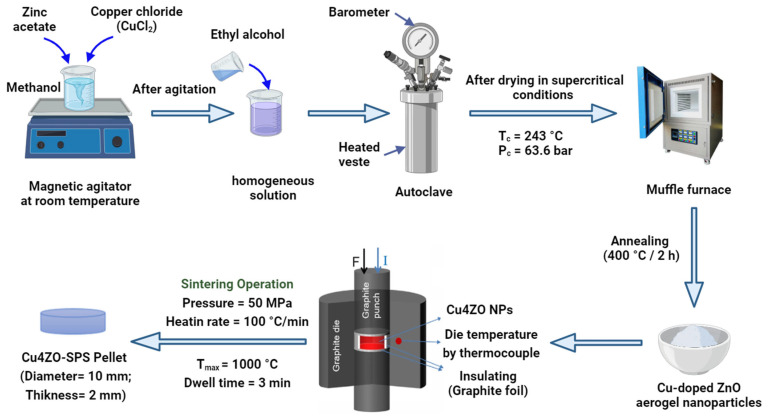
Preparation protocol using sol–gel method for Cu4ZO nanoparticles and SPS–Cu4ZO for pellet formation.

The synthesis of copper-doped zinc oxide (Cu4ZO) nanoparticles was carried out with meticulous precision using the sol–gel method. The process commenced by dissolving 16 g of zinc acetate dehydrate [Zn(CH_3_COO)_2_·2H_2_O; 99%] in 112 milliliters of methanol. This solution was then subjected to magnetic stirring at room temperature for a duration of 10 min. Following this, specific amounts of copper chloride (CuCl_2_) were carefully added to the solution to achieve a targeted [Cu]/[Zn] ratio of 0.04. After an additional 15 min of magnetic stirring to ensure thorough mixing, the resulting solution was transferred to an autoclave, where it underwent a crucial drying process under supercritical conditions, utilizing ethyl alcohol (EtOH). The use of supercritical drying is significant as it allows for the removal of the solvent in a manner that avoids the collapse of the nanoparticle structure, resulting in well-defined and highly porous nanopowders. Subsequently, the nanopowders obtained from the supercritical drying step underwent heat treatment in a furnace set at 400 °C for a duration of 2 h (Figure 1). This heat treatment process is essential for crystallization and the development of the desired structural and chemical properties in the synthesized nanoparticles. Notably, the heat treatment was conducted in an air environment, providing the necessary conditions for the desired phase transformation. To uniquely identify and reference the synthesized sample, it was assigned a distinct code, Cu4ZO.

### 2.2. Cu-Doped ZnO Ceramic Preparation by Spark Plasma Sintering

The Cu-doped ZnO (Cu4ZO) nanopowder underwent a precise Spark Plasma Sintering (SPS) process to transform it into a ceramic material with enhanced thermoelectric properties. This involved carefully positioning the Cu4ZO nanopowder within a graphite die, characterized by an inner diameter of 10 mm. The initiation of the sintering process was marked by the application of a maximum temperature of 1000 °C to all Cu4ZO nanoparticles. This temperature was achieved through a uniform heating rate of 100 °C/min. Crucially, a nitrogen gas atmosphere was maintained within the sintering chamber throughout the entire operation. The choice of atmosphere, in this case, is significant as it influences the chemical and thermal conditions during the sintering process, ensuring the desired properties in the resulting ceramic material. During the sintering operation, a uniaxial pressure of 50 MPa was consistently applied. The application of pressure is a key aspect of the SPS technique, as it aids in achieving high relative densities of the material, which is vital for enhancing its thermoelectric performance. The dwell time, representing the duration for which the specified temperature and pressure conditions are sustained, was carefully maintained at 3 min. This controlled dwell time is crucial in influencing the final properties of the ceramic material. The meticulous control over various parameters, including temperature, heating rate, gas atmosphere, pressure, and dwell time, played a pivotal role in optimizing the Spark Plasma Sintering technique for the synthesis of Cu4ZO ceramic. This optimization ensures uniformity and reproducibility in the resulting material’s characteristics, contributing to its reliability for a further analysis and application. The sintered samples took the form of 10-mm-diameter discs with a thickness of 2 mm, providing a standardized shape for subsequent characterization. To uniquely identify and reference the synthesized ceramic material, it was assigned a distinct code, SPS–Cu4ZO. More details are presented in Figure 1.

### 2.3. Characterizations

The characterization of the synthesized Cu-doped ZnO (SPS–Cu4ZO) material involved several advanced techniques to elucidate its structural, morphological, and electrical properties. For a detailed analysis of the crystalline structure, X-ray diffraction (XRD) was employed, utilizing a state-of-the-art instrument (XRD, D8 Advance, Bruker AXS, Karlsruhe, Germany). XRD is a powerful technique that provides information about the crystallographic phases present in the material. The resulting XRD patterns allowed for the identification and verification of the crystalline structure of the ZnO powder. To scrutinize the morphology and phase constitution at a microscale level, field-emission scanning electron microscopy (FEG-SEM) equipped with EDS was employed, utilizing the FEI Teneo instrument, manufactured by FEI Company in Hillsboro, OR, USA. This technique offers high-resolution imaging, enabling a detailed examination of the surface morphology and the distribution of different phases within the synthesized nanopowders. Impedance spectroscopy, a crucial aspect of electrical characterization, was conducted as a function of both temperature (ranging from 270 K to 370 K) and frequency (ranging from 100 Hz to 1 MHz). An Agilent 4294 analyzer (Agilent, Santa Clara, CA, USA) was utilized for these measurements, applying a voltage of 500 mV. The investigation involved the use of a prepared pressed pellet by SPS. Electrodes were deposited on opposite surfaces by coating them with a conductive silver paste. This approach allowed for the assessment of the electrical response of the Cu-doped ZnO ceramic under different temperature and frequency conditions.

## 3. Results and Discussion

### 3.1. Structural Properties

**Figure 2:**
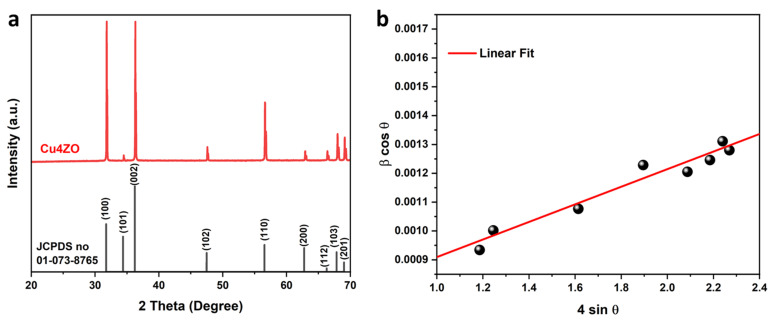
(**a**) X-ray diffractogram. (**b**) Williams–Hall plots of Cu4ZO ceramic prepared by SPS.

In Figure 2a, the XRD patterns provide crucial insights into the crystalline structure of both pure and copper-doped ZnO nanoparticles. The XRD patterns reveal the prevalence of the high hexagonal phase of ZnO. This hexagonal phase is a characteristic feature of well-crystallized ZnO structures. 

The XRD analysis highlights nine distinct diffraction peaks, each associated with specific reticular planes, which are indicative of the anisotropic growth observed in the synthesized samples. The identified peaks in the XRD patterns correspond to the reticular planes (100), (002), (101), (102), (110), (103), (200), (112), and (201). These peaks align precisely with the hexagonal crystal structure of ZnO, confirming the phase purity of the synthesized material. The assignment of these peaks is consistent with the hexagonal structure of ZnO, as documented in the JCPDS (Joint Committee on Powder Diffraction Standards) card No. 01-073-8765 [21]. Figure 2b illustrates Williamson–Hall plots, wherein micro-deformation (*ε*) and the average size of crystallites (*D*) were estimated utilizing the Williamson–Hall model [22]. The estimation process involved fitting the plots of (*βcosθ*) versus (4*sinθ*) according to the equation

(1) βcosθ=kλD+4 εsinθ

Here, *β* represents the integral breadth, *θ* is the Bragg angle, *k* is a constant, *λ* is the X-ray wavelength, *D* signifies the average crystallite size, and *ε* denotes the micro-deformation. The D value is determined from the slope of the linear fit extrapolation, and the ε value is obtained from the fit slope. The calculated average crystallite size was determined to be 229 ± 10 nm and the strain is around 0.00031 ± 0.00003. The structural parameters (*a*, *c*) for the samples were determined using the following equations:

(2) a=λ3sinθ(100), and c=λsinθ(002)

where *λ* is the wavelength of the radiation used (0.154 nm for the CuKα radiation), and *θ* is the Bragg diffraction angle. The derived values for (*a*, *c*) were (0.325 ± 0.001 nm, 0.521 ± 0.001 nm), corresponding to the structural parameters of hexagonal zinc oxide with lattice parameters of *a* = 0.3249 nm and *c* = 0.5206 nm [23]. 

### 3.2. Morphological and Chemical Composition Properties 

**Figure 3:**
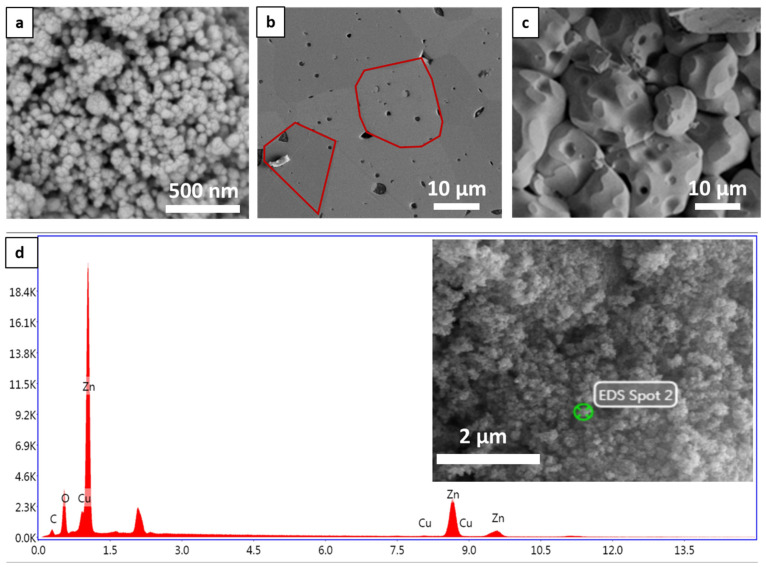
SEM images of (**a**) the prepared Cu4ZO nanoparticles and (**b**) the surface and (**c**) the fracture of the Cu-doped ZnO ceramic prepared by SPS. (**d**) EDS spectrum of the copper-doped ZnO nanoparticles prepared by sol–gel.

The morphological properties of the copper-doped ZnO nanoparticles were studied via SEM and are presented in Figure 3a. The analysis indicates that the nanoparticles exhibit a spherical shape with an average particle size of approximately 60 nm. Additionally, the morphological characteristics of the SPS–Cu4ZO pellet were thoroughly examined using SEM. 

Figure 3b,c present detailed views of the surface and fracture images, respectively, providing valuable insights into the structural characteristics of the prepared sample. In Figure 3b, the SEM images unveil a well-defined, compact structure characterized by an average particle size of 8.41 μm and the red boxes indicate the extremities of each particle in the structure, aiding in visualizing the particle boundaries. The high value of the average particle size in our structure and the small boundaries between each particle are contributors to a profound effect on enhancing electron transport. Larger particle sizes can facilitate better electron transport within the material, potentially improving its electrical conductivity. Delving deeper into the structure’s interior in Figure 3c, the SEM analysis reveals the existence of pores with dimensions ranging from 0.5 to 3 μm. The presence of pores is a crucial aspect of the material’s morphology, influencing properties such as electrical conductivity and porosity. The observed pore dimensions suggest a porous network within the material, which can contribute to an increase in electrical conductivity. Controlling and understanding the distribution of these pores is essential for tailoring the thermoelectric properties of the material, as it can impact both electrical and thermal transport. The chemical composition of the Cu-doped ZnO sample was studied by the EDS spectrum presented in Figure 3d. It revealed the presence of base elements of zinc oxide, such as zinc and oxygen, along with carbon elements from the precursor used for preparation. In addition to proving the presence of the doping element, we confirm the presence of copper (Cu) in the structure of the prepared sample.

### 3.3. Electrical Behaviors

#### 3.3.1. Electrical Conductivity

**Figure 4:**
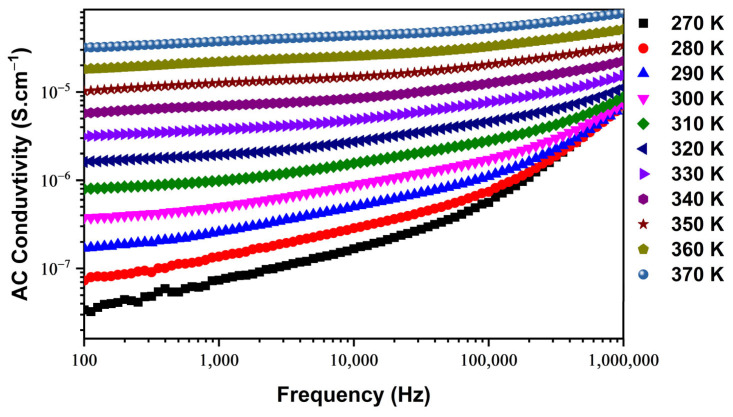
ac conductivity dependence of frequency as a function of different temperatures.

Analyzing changes in conductivity as a function of alternating current (ac) frequency provides valuable insights into the charge transport mechanism and interactions among charge carriers within a material. In Figure 4, we observe the variation of this physical property concerning angular frequency at different temperatures. The presented conductivity spectra offer a nuanced understanding of the electrical behavior of the material under varying frequency conditions. The conductivity spectra depicted in Figure 4 reveal the presence of two distinct contributions. At low frequencies, the conductivity is attributed to grain boundaries, while at high frequencies, it is associated with the grains themselves. This dual contribution reflects the complex nature of charge transport mechanisms within the Cu-doped ZnO material. In the low-frequency range, the conductivity exhibits a consistent and uniform profile that progressively increases with temperature. This observation suggests the activation of thermal conduction processes within the material. The consistent and uniform conductivity profile at low frequencies is indicative of a predominant role played by grain boundaries in facilitating charge transport [24,25].

The increase in conductivity with temperature further implies the enhancement in electrical transport processes, which is a common behavior in materials undergoing thermal activation. Understanding the distinct contributions to conductivity at different frequencies is crucial for tailoring the material’s properties for specific applications. The identified roles of grain boundaries and grains in charge transport provide insights into the electrical behavior of the Cu-doped ZnO material under dynamic conditions. To comprehensively model the high-frequency spectra depicted in Figure 4, we employ Jonscher’s universal law, expressed as follows [26,27]:

(3) $$\sigma_{ac}\left(\omega \right)=\sigma_{dc}+{A\omega }^{s}$$

In the given context, *ω* signifies pulsation, *σ_dc_* represents dc conductivity, *A* is a constant dependent on temperature, and (0 ≤ *s* ≤ 1) is a dimensionless parameter that describes dispersion within the material. This parameter plays a crucial role in comprehending the conduction properties of the sample. 

**Figure 5:**
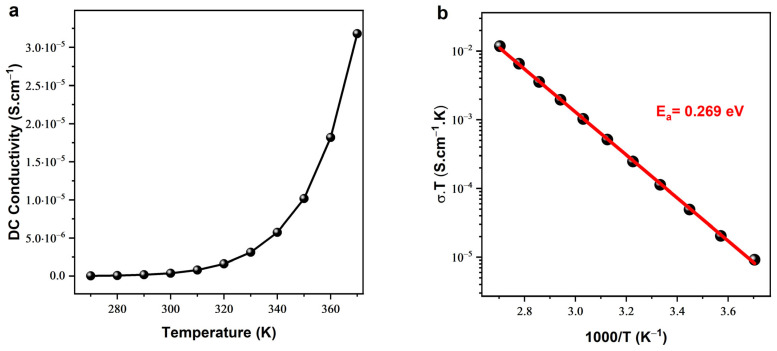
(**a**) Temperature dependence of dc conductivity. (**b**) Semi-logarithmic σ×T vs. 1000/T plot.

The temperature dependency of dc conductivity, as illustrated in Figure 5a, examined reveals an exponential correlation with temperature in accordance with the Arrhenius law. The Arrhenius law is expressed as follows [28,29]:

(4) σdc=A×exp−EakBT

where *E_a_* is the activation energy of the moving charge carriers, *A* is a constant, and *k_B_* is the Boltzmann constant. 

To estimate the activation energy *E_a_*, the semi-logarithmic curve of *σ_dc_×T* as a function of 1000/T was plotted, as shown in Figure 5b. *E_a_* was determined by extracting the slope of the linear fit from the traced curve, resulting in a value of 0.269 ± 0.021 eV. The elevated value of the activation energy signifies that our prepared Cu-doped ZnO in the ceramic dense state possesses distinctive characteristics. This high activation energy has profound implications, particularly in thermoelectric applications, as it indicates a substantial energy barrier for charge carriers [30]. Understanding and utilizing materials with high activation energy is crucial in enhancing thermoelectric efficiency, as it hinders thermal diffusion and promotes efficient electrical conduction, making the material promising for thermoelectric device applications.

#### 3.3.2. Electrical Impedance

##### Real Part of Impedance

**Figure 6:**
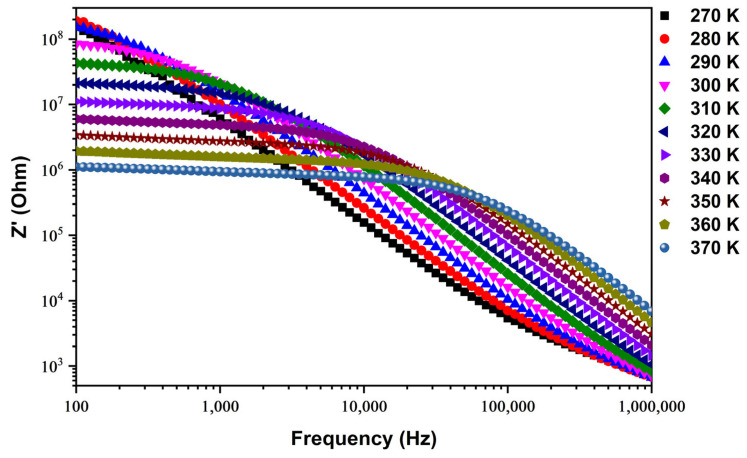
Frequency dependence of the real part of impedance (*Z*′) in wide range of temperatures.

Figure 6 provides a comprehensive representation of the variation in the real part of impedance (*Z*′) for the SPS–Cu4ZO ceramic across a wide spectrum of frequencies and temperatures. The observed trends in *Z*′ offer valuable insights into the electrical characteristics of the synthesized material, contributing to a deeper understanding of its thermoelectric performance. Our findings reveal a noteworthy behavior in the *Z*′ values at lower frequencies, where a consistent value is maintained. As the frequency increases, a systematic decrease in *Z*′ is observed, signaling an enhancement in the material’s conductivity. This frequency-dependent response aligns with the typical behavior of conductive materials, where higher frequencies promote greater ease of electron movement, leading to improved conductivity [31,32]. Additionally, the *Z*′ values exhibit temperature-dependent fluctuations, with a convergence observed at higher frequencies. This behavior is indicative of semiconductor characteristics in the material. The diminishing trend of *Z*′ with increasing temperature further reinforces the semiconductor nature of the composition. This phenomenon is consistent with the behavior of semiconductors, where elevated temperatures can lead to a reduction in electrical resistivity. At elevated frequencies, the *Z*′ values exhibit a merging pattern, a phenomenon that can be attributed to the release of space charge and diminished barrier properties within the material. This observation aligns with existing literature on diverse materials [33,34,35] and suggests that, at higher frequencies, the electrical properties of the SPS–Cu4ZO ceramic may be influenced by factors such as charge carriers and barrier effects.

##### Imaginary Part of Impedance

**Figure 7:**
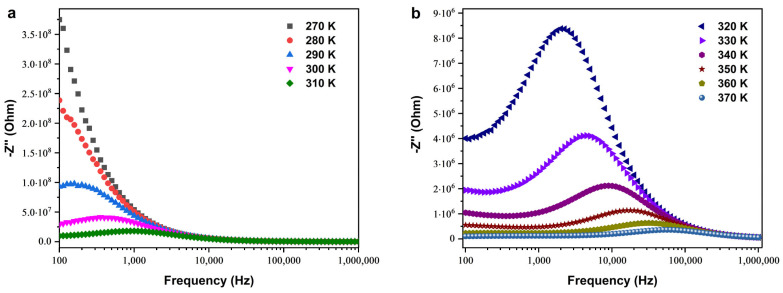
Frequency dependence of the imaginary part of impedance (*Z*″) in two ranges of temperatures, (**a**) from 270 to 310 K and (**b**) from 320 to 370 K.

The analysis of the imaginary impedance (*Z*″) of the SPS–Cu4ZO sample is presented in Figure 7, providing valuable insights into the temperature-dependent electrical behavior of the synthesized material. The *Z*″ spectrum is plotted as a function of frequency, revealing distinct patterns that shed light on the electrical characteristics of the Cu-doped ZnO ceramic. Notably, the *Z*″ spectrum exhibits prominent peaks at lower temperatures. As the temperature increases, these peaks undergo a noticeable transformation, becoming more flattened. This observation suggests a widening or broadening of the peaks with rising temperatures. In practical terms, this indicates that the electrical response of the material undergoes changes as the temperature increases, leading to a more dispersed distribution of energy across a range of frequencies. Furthermore, the maximum *Z*″, corresponding to the peak values, shifts toward higher frequencies as the temperature increases. This shift implies an increase in the loss tangent, indicating a temperature-dependent electrical relaxation phenomenon within the Cu-doped ZnO material. In simpler terms, the electrical behavior of the material experiences alterations in response to temperature variations, a phenomenon that manifests in the observed shifts and broadening of peaks in the *Z*″ spectrum [36,37,38]. This temperature-dependent electrical relaxation is a critical aspect of understanding the dynamic response of the material under different thermal conditions. The observed changes in the *Z*″ spectrum highlight the complex interplay between temperature and the electrical properties of the Cu-doped ZnO ceramic. Such insights are essential for unraveling the material’s behavior in practical applications, especially in the thermoelectric realm, where a nuanced understanding of electrical characteristics under varying conditions is crucial. The findings presented in Figure 7 contribute significantly to advancing our understanding of the Cu-doped ZnO material and pave the way for its targeted optimization for specific thermoelectric applications.

The compound’s tendency to relax and its polarization, which are identified as space charges, are apparent as the relaxation frequency (*F_max_*) rises and the relaxation time (*τ*) diminishes with increasing temperature. The temperature-dependent behavior of the relaxation frequency (*F_max_*) can be described using the Arrhenius relation, expressed as follows [39,40]:

(5) Fmax=f0 exp −EakBT

**Figure 8:**
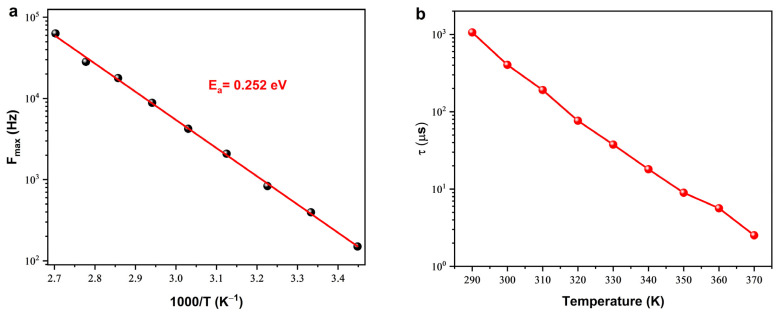
(**a**) Frequency-max and (**b**) relaxation time (*τ*) variation as a function of temperature.

The symbols $f_{0}$, $E_{a}$, and $k_{B}$ correspond to the pre-exponential term, activation energy, and Boltzmann constant, respectively. In Figure 8a, the semi-logarithmic variation of *F_max_* is presented as a function of 1000/T, revealing an activation energy (*E_a_*) value of 0.252 ± 0.041 eV derived from the linear fit of the curve. This value is close to the one estimated from the curve of the temperature dependence of the DC conductivity (*E_a_* = 0.269 ± 0.021 eV).

The relaxation time (*τ*) of the SPS–Cu4ZO structure, a key parameter characterizing the response of charge carriers to an applied electric field, can be determined using the equation *τ* = 1/(2*πF_max_*) [41]. Figure 8b represents the relationship between the relaxation time and temperature, revealing an intriguing pattern that has significant implications for the electrical behavior of the material. 

As temperature increases, the relaxation time (*τ*) decreases. This observation carries profound physical implications. The relaxation time is associated with the time it takes for electrical charges to return to their equilibrium positions after being subjected to an external perturbation [42]. In the context of the Cu-doped ZnO material, this perturbation could be induced by thermal fluctuations or the application of an electric field. A decrease in relaxation time with increasing temperature implies that the orientation of electrical charges in the interface is facilitated by heating. In other words, higher temperatures contribute to a more rapid realignment of charge carriers in response to external influences. This phenomenon is particularly crucial in the context of charge transport within the material. A lower relaxation time (*τ*) under high-temperature conditions indicates more efficient exciton dissociation at the structure [43,44]. Exciton dissociation is the phenomenon wherein an excited electron and hole pair, collectively termed an exciton, undergoes separation into free charge carriers. The heightened responsiveness of the structure, attributed to a faster charge transport process and a lower relaxation time, underscores its excellent performance. Practically, this implies that, particularly under elevated temperatures, the Cu-doped ZnO material exhibits an enhanced capability to swiftly respond to external stimuli, a highly desirable trait for various electronic and optoelectronic applications. The observed decrease in relaxation time with increasing temperature in the SPS–Cu4ZO structure suggests improved charge carrier mobility and exciton dissociation efficiency. The thermal transport and thermoelectric characteristics of doped ZnO are intricate, influenced by diverse factors, including temperature and electron relaxation time. Our investigation specifically delved into doping ZnO with Cu to tailor its electrical, thermal properties, and thermoelectric performance. In Cu-doped ZnO, both electronic and phononic contributions play pivotal roles in affecting thermal conductivity, with electron–phonon scattering emerging as a critical process. Furthermore, as temperature rises, phonons and electron movements gain more energy, resulting in a reduction in electron relaxation time. The interplay among these factors significantly shapes the material’s thermal properties. This comprehensive understanding is imperative for optimizing the material’s performance in applications where a rapid and efficient response to external influences is crucial, such as in thermoelectric devices and sensors [45].

##### Nyquist Diagrams

**Figure 9:**
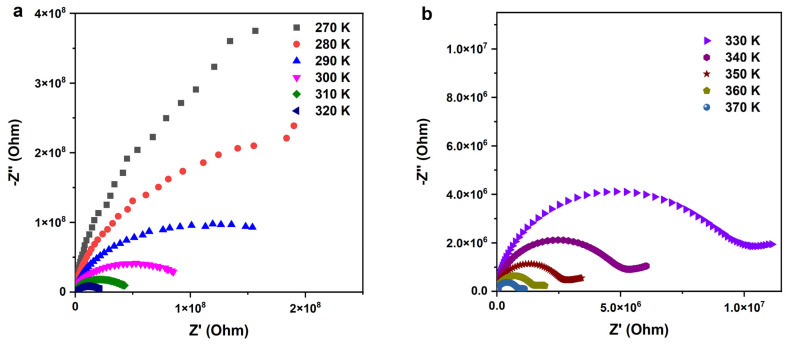
Nyquist diagrams of the SPS–Cu4ZO sample in wide temperature ranges, (**a**) from 270 to 320 K, and (**b**) from 330 to 370 K.

The Nyquist plot depicted in Figure 9 provides a detailed visualization of the intricate relationship between the real part (*Z*′) and imaginary part (*Z*″) of the impedance across a broad frequency spectrum ranging from 100 Hz to 1 MHz and temperatures spanning from 270 K to 370 K. This plot is instrumental in unraveling the conduction mechanisms and dynamic electrical behavior of the Cu-doped ZnO (SPS–Cu4ZO) ceramic under varying conditions. The impedance spectra manifest as distinct semicircular arcs at all temperatures, indicative of grain boundary conduction predominantly dictating the conduction process within the samples. The semicircular arc pattern is characteristic of materials with a distribution of relaxation times, commonly associated with grain boundaries. As the temperature increases, there is a noticeable reduction in the radius of these semicircular arcs, coupled with a discernible shift in their center points toward the axis center. This shift suggests the presence of a relaxation time distribution and implies a non-Debye-type relaxation mechanism within the examined structure [46,47]. The nuanced changes in the Nyquist plot as a function of temperature offer valuable insights into the dynamic electrical response of the material, providing a basis for understanding its behavior under varying thermal conditions. In the elevated temperature range, specifically from 330 K to 370 K (as illustrated in Figure 9b), the Nyquist diagram reveals the presence of two distinct semicircles. The first, observed at higher frequencies, is contingent upon the presence of grain, signifying the influence of grain boundaries on the conduction process. The second semicircle, apparent at lower frequencies, is attributed to the electrode effect [48]. This dual semicircle structure further underscores the complexity of the conduction mechanisms in the Cu-doped ZnO ceramic, with different processes contributing to the overall impedance response.

##### Electrical Modulus

The complex electric modulus, denoted as M*, is the reciprocal of the complex permittivity and is expressed mathematically as M* = M′ + jM″ [49], with M′ and M″ representing the real and imaginary components, respectively. The angular frequency (ω = 2πf) is associated with the applied AC frequency.

**Figure 10:**
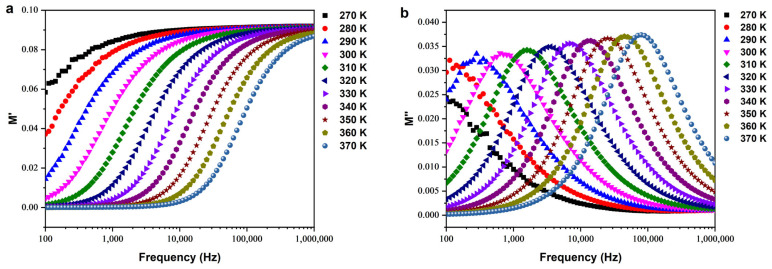
(**a**) Real part and (**b**) imaginary part of the electrical modulus.

In Figure 10a, the impact of the real part of the modulus (M′) is depicted across a temperature range of 270–370 K as a function of frequency. At lower frequencies, M′ approaches zero, indicating reduced responsiveness to the applied electric field, attributed to slower relaxation processes like ionic migration or dipolar reorientation. With increasing frequency and temperature, M′ values rise, indicating an improved material response linked to accelerated relaxation processes [50].

Figure 10b illustrates the variation of the imaginary part of the modulus (M″) with frequency, providing insights into energy dissipation mechanisms within the ceramic. Analyzing peak positions and slopes in the M″ plot offers a precise understanding of relaxation processes. Peaks in the M″ plots indicate relaxor behavior, contributing to energy conservation, confirmed by symmetric shifts in the peaks. The observed behavior suggests the presence of a non-Debye relaxation mechanism within the sample [51].

### 3.4. Dielectric Behaviors

**Figure 11:**
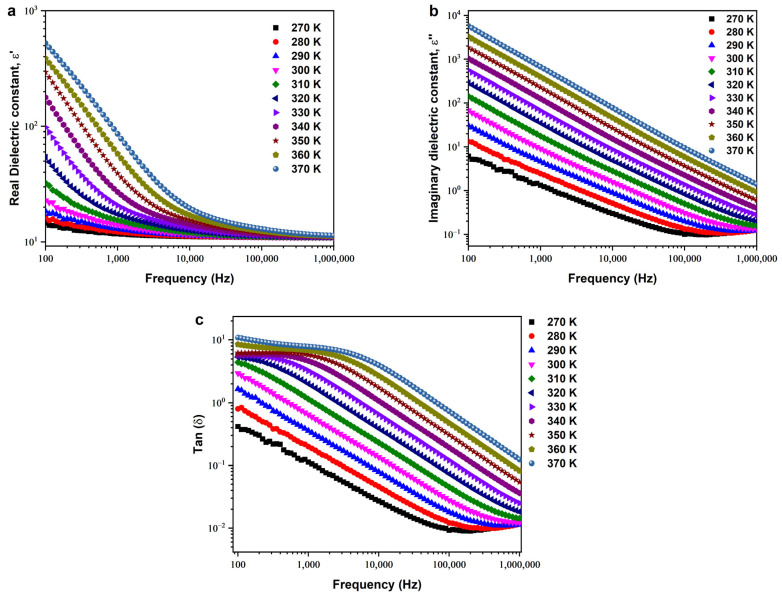
(**a**) Real part and (**b**) imaginary part of the dielectric constant as a function of frequency. (**c**) Dielectric loss of the prepared SPS–Cu4ZO sample.

In Figure 11a, the real part (ε′) of the permittivity is presented against the frequency, spanning temperatures from 270 K to 370 K in 10 K intervals. The observed trend indicates an increase in permittivity with temperature, showcasing the material’s sensitivity to thermal variations. However, as the frequency escalates, there is a notable decrease in permittivity, suggesting a frequency-dependent response. This behavior can be attributed to the complex interplay of dipolar and ionic interactions within the copper-doped ZnO material [52].

Moving to Figure 11b, the imaginary part of the permittivity (ε″) is depicted at different temperatures as a function of frequency. At low frequencies and elevated temperatures, ε″ exhibits a substantial increase. This phenomenon can be elucidated by considering the accumulation of charges at the electrode–compound interface. The elevated ε″ values may result from ion diffusion effects, indicating a non-Debye-type behavior in the material. This intricate behavior reflects the material’s response to varying external conditions, offering insights into its charge storage and transport mechanisms [53].

As temperature rises, Figure 11c illustrates an augmentation in dielectric losses with a notable prominence at low frequencies and high temperatures. This behavior aligns with the improved conductivity of the copper-doped ZnO material at elevated temperatures. The increased dielectric constant is indicative of enhanced charge carrier mobility, contributing to the material’s ability to respond to an external electric field [54].

## 4. Conclusions

In conclusion, the synthesis of copper-doped zinc oxide (Cu4ZO) nanoparticles and ceramics through a controlled sol–gel method and SPS technique, respectively, has been successfully achieved. The structural analysis revealed a hexagonal ZnO phase, while morphological assessments depicted a well-defined compact structure with intrinsic pores. Electrical characterization through impedance spectroscopy demonstrated the material’s unique electrical behaviors, with distinct contributions from grain boundaries and grains themselves. The activation energy of 0.269 eV signifies the potential of the Cu4ZO ceramic for thermoelectric applications due to its substantial energy barrier for charge carriers. The complex impedance spectroscopy further unveiled temperature-dependent electrical relaxation phenomena, affirming the material’s versatility. The Nyquist diagrams illustrate the dominance of grain boundary conduction, indicating the potential applicability of the material in various electronic devices. The dielectric behaviors exhibit low dielectric loss. This study provides valuable insights into the synthesis and characterization of Cu-doped ZnO, offering a foundation for further exploration in diverse technological applications.

## Data Availability

The data are available upon request from the corresponding author.
